# Long non-coding RNA Peg13 attenuates the sevoflurane toxicity against neural stem cells by sponging microRNA-128-3p to preserve Sox13 expression

**DOI:** 10.1371/journal.pone.0243644

**Published:** 2020-12-09

**Authors:** Yunfeng Jiang, Yue Wang, Yu Sun, Hong Jiang

**Affiliations:** Department of Anesthesiology, Shanghai Ninth People’s Hospital, Shanghai Jiao Tong University School of Medicine, Center for Specialty Strategy Research of Shanghai Jiao Tong University China Hospital Development Institute, Shanghai, China; University of Tennessee Health Science Center College of Medicine Memphis, UNITED STATES

## Abstract

**Background:**

Exposure to anesthetics during brain development may impair neurological function, however, the mechanisms underlying anesthetic neurotoxicity are unclear. Recent studies indicate that long non-coding RNAs (lncRNAs) are crucial for regulating the functional brain development during neurogenesis. This study aimed to determine the regulatory effects and potential mechanisms of lncRNA Peg13 (Peg13) on sevoflurane exposure-related neurotoxicity against neural stem cells (NSCs).

**Methods:**

Mouse embryotic NSCs were isolated and their self-renewal and differentiation were characterized by immunofluorescence. NSCs were exposed to 4.1% sevoflurane 2 h daily for three consecutive days. The potential toxicities of sevoflurane against NSCs were evaluated by neurosphere formation, 5-ethynyl-2'-deoxyuridine (EdU) incorporation and flow cytometry assays. The Peg13, miR-128-3p and Sox13 expression in NSCs were quantified. The potential interactions among Peg13, miR-128-3p and Sox13 were analyzed by luciferase reporter assay. The effects of Peg13 and/or miR-128-3p over-expression on the sevoflurane-related neurotoxicity and Sox13 expression were determined in NSCs.

**Results:**

The isolated mouse embryotic NSCs displayed potent self-renewal ability and differentiated into neurons, astrocytes and oligodendrocytes in vitro, which were significantly inhibited by sevoflurane exposure. Sevoflurane exposure significantly down-regulated Peg13 and Sox13, but enhanced miR-128-3p expression in NSCs. Transfection with miR-128-3p mimics, but not the control, significantly mitigated the Peg13 or Sox13-regulated luciferase expression in 293T cells. Peg13 over-expression significantly reduced the sevoflurane-related neurotoxicity and increased Sox13 expression in NSCs, which were mitigated by miR-128-3p transfection.

**Conclusion:**

Such data indicated that Peg13 mitigated the sevoflurane-related neurotoxicity by sponging miR-128-3p to preserve Sox13 expression in NSCs.

## Introduction

The focus on neurotoxicity of anesthesia in early life remains unabated [1–3]. Several clinical studies have reported that anesthesia and surgery in early life are associated with neuropsychological and behavioral defects in children. A recent study has shown that repeated exposure to anesthesia can impair the processing speed and fine motor abilities, but does not significantly affect intelligence in children [4]. Similarly, multiple exposures to anesthesia in young animals can result in neuroinflammation [5, 6], synaptic dysfunction [7, 8], impair motor [4] and behavioral development and stress responses [9]. Our previous studies have found that sevoflurane exposure can damage the myelin sheath in the developing brain of mice and their cognitive capacity [10] and impair the differentiation of embryonic stem cells (ESCs) [11]. However, whether sevoflurane can modulate the self-renewal and differentiation of neural stem cells (NSCs) has not been explored.

The precise control of the self-renewal and differentiation of NSCs is crucial for the brain development and function [12–14]. Their disruption may cause microcephaly or megalencephaly, leading to severe birth defects and life disability [15]. NSCs have potent self-renewal ability to form neurospheres and can differentiate into β-tubulin-III (Tuj1) + neurons, O4+ oligodendrocytes, and GFAP+ astrocytes [16, 17]. Previous studies have shown that anesthesia, such as propofol, ketamine and isoflurane, can inhibit the self-renewal and differentiation of mouse NSCs [18, 19]. Sevoflurane is commonly used in pediatric and obstetric anesthesia, and it is important to understand its potential neurotoxicity and mechanisms in NSCs. Previous studies have reported that sevoflurane can inhibit the proliferation and differentiation of hippocampal NSCs, which is association with up-regulating miR-183 to down-regulate NR4A2 expression or inhibiting the Let-7a-Lin28 signaling [20, 21]. However, the mechanisms by which sevoflurane affects the proliferation and differentiation of NSCs have not been fully elucidated.

Long non-coding RNAs (LncRNAs) can regulate many biological processes during the development, such as neuronal development, plasticity, disease, and evolution [22, 23]. LncRNA Pnky can regulate the neuronal differentiation of NSCs [24] and lncRNA Gadd45a, Rik-203 and WNT5A-AS participate in the neurotoxicity of sevoflurane anesthesia [11, 25, 26]. LncRNA Peg13, a new lncRNA, may be involved in the regulation of mate selection specific to the mouse population [27] and its expression is significantly down-regulated in primary brain microvascular endothelial cells after oxygen-glucose deprivation in vitro [28]. However, there is no information on whether and how lncRNA Peg13 regulates the sevoflurane-related neurotoxicity against NSCs.

This study aimed (1) to determine the impact of multiple sevoflurane exposure on the self-renewal and differentiation of mouse NSCs; and (2) to reveal the potential mechanisms underlying the regulatory role of Peg13 in sevoflurane-related neurotoxicity against NSCs in vitro.

## Materials and methods

### Establishment of neural stem cell differentiation in vitro

The experimental protocols were approved by the Animal Care and Use Committee of the Ninth People's Hospital in Medical School of Shanghai Jiao Tong University. The NSCs were isolated from C57BL/6 mice at E14.5 and grown in DMEM-F12 (1:1) maintenance medium containing 20 ng/mL of human epidermal growth factor (hEGF), basic fibroblast growth factor (bFGF), 1% N2, 2% B27 (Gibco, New York, USA), as described previously [29]. The cloned NSCs (6 x 10^5^ cells/well) were cultured onto coverslips that had been coated with poly-l-lysine/laminin (Invitrogen, Carlsbad, USA) in DMEM-F12 (1:1) differentiation medium containing half concentration of growth factors to induce their differentiation for subsequent experiments.

### Sevoflurane exposure

NSCs were exposed to sevoflurane, as described previously [10]. Briefly, the cells were cultured in 60% O_2_ or 4.1% sevoflurane and 60% O_2_ (1 L/min) for 2 h daily for three consecutive days. The RNAs and proteins were extracted immediately after sevoflurane exposure for subsequent experiments.

### Neurosphere assay

**Fig 1:**
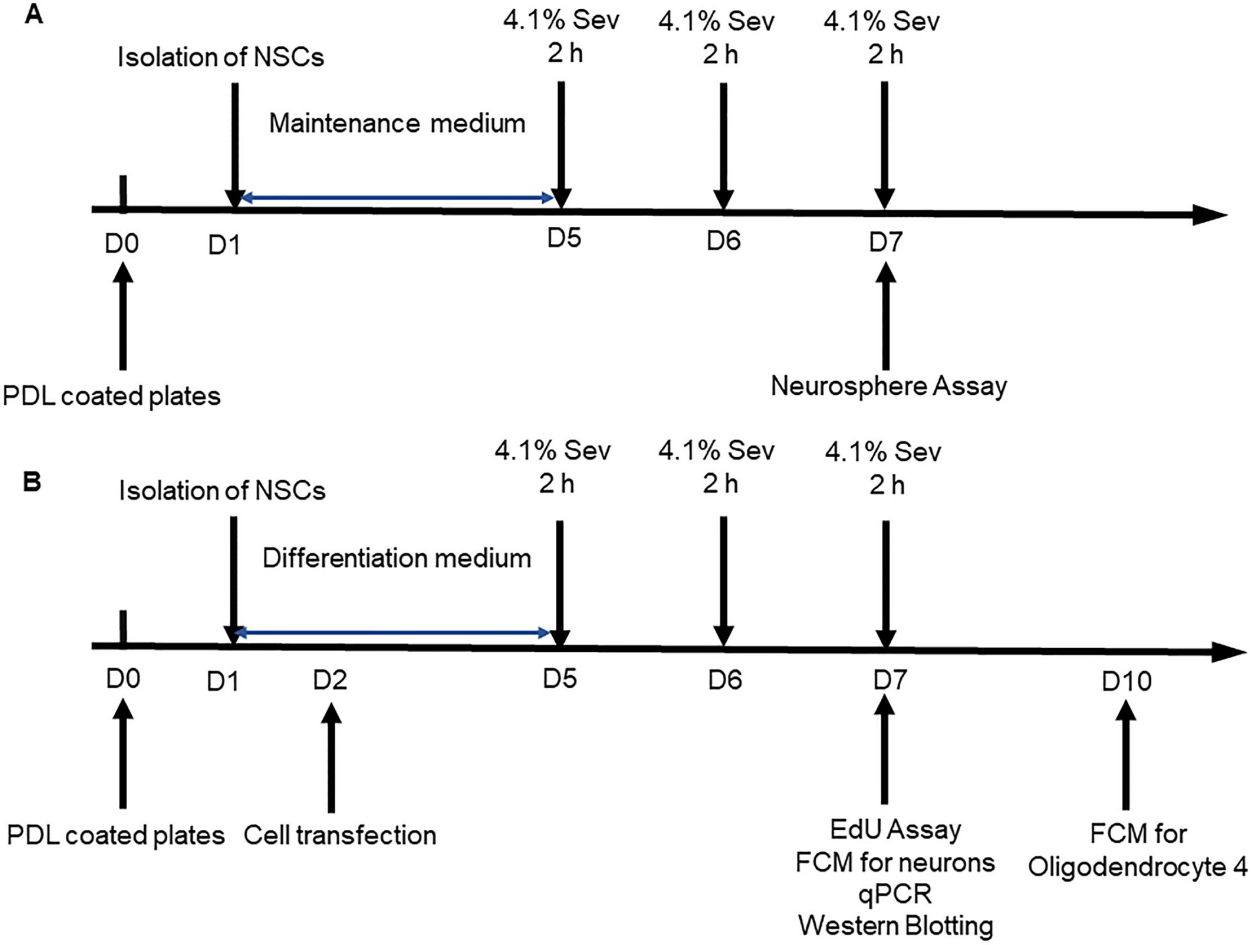
Diagrams for the experimental design. A. NSCs were cultured in DMEM-F12 (1:1) maintenance medium containing 20 ng/mL of hEGF, bFGF, 1% N2 and 2% B27 for four days. The NSCs were exposed to sevoflurane (4.1% sevoflurane in 60% O_2_) or control 60% O_2_ alone 2 h daily for three consecutive days. The self-renewal of NSCs was analyzed by neurosphere formation. B. The NSCs (6 x 10^5^ cells/well) were cultured onto coverslips that had been coated with poly-l-lysine/laminin in DMEM-F12 (1:1) differentiation medium containing half concentration of growth factors to induce their differentiation for four days. The NSCs were exposed to sevoflurane (4.1% sevoflurane in 60% O_2_) or control 60% O_2_ alone 2 h daily for three consecutive days. The differential neurons and oligodendrocytes and astrocytes were characterized at day 7 and 10 post induction, respectively.

NSCs (5x10^4^ cells/well) were cultured in 24-well plates for 4 days in DMEM-F12 (1:1) maintenance medium and exposed to sevoflurane daily for three consecutive days (Fig 1A). The images of neurospheres were captured under a light microscope (Leica, Germany). The diameter of neurospheres was measured using ImageJ software (NIH, Bethesda, USA).

### EdU assay

The effect of sevoflurane on the proliferation of NSCs was determined by EdU assay using a BeyoClick™EdU‐595 kit (Beyotime, Beijing, China), according to the manufacturer’s instruction. Briefly, NSCs were cultured in differentiation medium for 4 days and exposed to 60% O_2_ alone (control) or 60% O_2_ and sevoflurane as described in the Fig 1B. The cells were cultured in differentiation medium containing 10 μM EdU (EdU medium) for 12 h. Subsequently, the cells were fixed in 4% paraformaldehyde and incubated with 3% bovine serum albumin (BSA) and 0.3% Triton X-100 (Sigma, St. Louis, USA) in phosphate buffered saline (PBS). The cells were stained with a Click Additive Solution provided in the kit and nuclearly stained with DAPI (1:1000, Sigma). The images were taken under a fluorescent microscope (Leica, Germany), and the fluorescent signals were analyzed by ImageJ software. Finally, the percentages of EdU+ cells were analyzed.

### Immunofluorescence

NSCs and their differentiated cells were cultured onto coverslips for 3 days, fixed with 4.1% paraformaldehyde, permeabilized with 0.3% Triton X-100 and blocked with 5% BSA. The cells were incubated with primary antibodies against GFAP, Tuj1 (1:200, abcam, Cambridge, UK), Oligodendrocyte Marker O4 (1:200, R&D, Minneapolis, USA) and Nestin (1:200, Sigma) at 4°C overnight. The same concentrations of mouse and rabbit IgG from unimmunized animals (1:1000, Cell Signaling Technology, Beverly, USA) were used as negative controls. After being washed for 3 times, the cells were incubated with secondary antibodies including Alexa Fluor 488-conjugated donkey anti-mouse and Alexa Fluor 574-labeled donkey anti-rabbit IgG (1:1000, Cell Signaling Technology). The nuclei were stained with DAPI (1mg/ml, 1:1000). The fluorescent signals were observed under a fluorescent microscope and analyzed by ImageJ software.

### Total RNA extraction for RNA sequencing

NSCs were cultured in the differentiation medium and exposed, or not (control), to sevoflurane for three days. Their RNAs were extracted using Trizol reagent (Invitrogen) and RNeasy mini kit (Qiagen, Valencia, USA), according to the manufacturer’s protocol. The quality and quantity of RNA samples were measured by NanoDrop ND-8000 (ThermoFisher Scientific, Waltham, USA). The RNA fragmentation, purification, terminus modification, cDNA library generation, sequencing, and data analyses were performed as described previously [30]. Briefly, after removal of ribosomal RNA, the RNA samples were fragmented and reversely transcribed into cDNA. The cDNA was purified and individual DNA fragments were added with adenylate at the 3’end, followed by ligating adapters. The cDNA fragments were further purified and enriched with PCR to create the final cDNA libraries. The cDNA libraries were sequenced in HiSeq 4000 (Illumina, San Diego, USA). The differentially expressed genes (DEGs) were determined using the Cuffdiff, based on a |log2-fold change (FC)| >1.5 [31]. The potential multiple testing-related false positives were adjusted by false discovery rate (FDR) test and FDR < 0.05 was considered statistically significant.

### Transfection

To induce Peg13 overexpression, the DNA fragment for Peg13 expression was cloned into plasmid pGMLV-CMV-MCS-EF1-ZsGreen1-T2A-Puro to get pGMLV-CMV-Peg13-EF1-ZsGreen1-T2A-Puro plasmid, which was sequenced by Genomeditech (Shanghai, China). Next, 293T cells were transfected with 3 μg pGMLV-CMV-Peg13-EF1-ZsGreen1-T2A-Puro or vehicle plasmid, 3 μg pMD2.D and 6.0 μg pSPAX2 using lipofectamine 3000 reagent (Invitrogen) to generate control lentivirus (OE-NC) or lentivirus for Peg13 expression (OE-Peg13), as described previously [32].

NSCs (6×10 ^5^ cells/well) were cultured overnight in six-well plates and transduced with control lentivirus (OE-NC) or lentivirus (OE-Peg13) at a MOI of 10 for inducing Peg13 over-expression. The cells were treated with 4 μg/ml puromycin for 7 days to establish stable Peg13 overexpressing cells. In addition, NSCs were transfected with 50 nM miR-128-3p mimic or scrambled control (RIBOBIO, Guangzhou, China) using the riboFECT™ CP (RIBOBIO). Their transfection efficiency was determined by qRT-PCR.

### Western blotting

The impact of repeated sevoflurane exposure on the expression of Sox13 in NSCs was determined by Western blot assay. Briefly, NSCs were cultured in the differentiation medium and exposed to 4.1% sevoflurane and 60% O_2_ (1 L/min), or 60% O_2_ alone, for 2 h daily for three consecutive days. The cells were harvested and lyzed in RIPA (Radio Immunoprecipitation Assay) buffer (Beyotime). After determination of protein concentrations, the cell lysates (50 μg/lane) were separated by SDS-PAGE on 8% gels and transferred onto polyvinylidene difluoride (PVDF) membranes (Millipore, Billerica, USA). The membranes were blocked with 5% skim milk in TBST, and incubated with primary antibodies against Sox13 (1:1000, Santa Cruz Biotechnology, CA, USA) and β-actin (1:1000, Cell Signaling Technology) at 4°C overnight. The bound antibodies were detected with HRP-conjugated anti-mouse IgG (1:10000, Cell Signaling Technology) and visualized using the enhanced chemiluminescence (ECL, ThermoFisher Scientific). The relative levels of the Sox13 to control β-actin expression were quantified by ImageJ Software.

### Flow cytometry

The NSCs (6 x 10^5^ cells/well) were cultured in DMEM-F12 (1:1) differentiation medium to induce their differentiation for four days. The NSCs were exposed to sevoflurane (4.1% sevoflurane in 60% O_2_) or control 60% O_2_ alone 2 h daily for three consecutive days. The neuronal and oligodendrocyte differentiation was detected at day 7 and 10 post induction by indirect immunofluorescent assays, respectively. The cells were stained with anti-Tuj1 (1:200, abcam), and anti-O4 (1:200, R&D, Minneapolis, USA) or control rabbit or mouse IgG. After being washed, the cells were reacted with Alexa Fluor 488-conjugated donkey anti-mouse IgG or donkey anti-rabbit IgG. The unstained cells served the controls. The cells were analyzed by flow cytometry in a BD FACSCanto (BD Biosciences, New Jersey, USA). The data were analyzed by Flowjo 10 software.

### Quantitative real-time PCR (qRT-PCR)

After repeated exposure of NSCs to sevoflurane, the relative levels of Peg13, Sox13 mRNA and relevant miRNA transcripts to U6 or GAPDH in different groups of cells were quantified by qRT-PCR using specific primers, the SYBR Green I Real-Time PCR kit (Takara), or miDETECT A TrackTM miRNA qRT-PCR Starter Kit (RIBOBIO), respectively. The data were analyzed by the 2^−ΔΔCt^ method. The sequences of primers were Peg13 forward 5'-CTCACTTTGGTTTGAATGGGAT-3', and reverse 5'-AAGACGATTAGATTGGGTTGC-3'; Sox13 forward 5'-AGCAAGATCCTTGGTTCTCG-3', and reverse 5'-GGAGACTGCAGGTATTGATG-3'; GAPDH forward 5'-AATGGATTTGGACGCATTGGT-3' and reverse 5'-TTTGCACTGGTACGTGTTGAT-3'. The primers for miRNA and U6 were purchased from RIBOBIO.

### Agarose gel electrophoresis

Total RNA was extracted from the brain, liver, lung, heart, limb and kidney of E17.5 embryonic mice using Trizol reagent (Takara) and reversely transcribed into cDNA using a cDNA Synthesis Kit (Takara). The levels of Peg13 transcripts were semi-quantified by PCR using specific primers Peg13 (PF: 5'-CTCACTTTGGTTTGAATGGGAT-3', and RF:5'-AAGACGATTAGATTGGGTTGC-3'). The PCR products and DNA markers were analyzed by agarose gel electrophoresis using 2% agarose gels (BioRad, CA, USA) and visualized in the gel imager (ABI, CA, USA).

### Fluorescence in situ hybridization (FISH)

The differentiated neuronal cells were cultured on a PDL-coated glass slide for 3 days. The distribution of Peg13 in the cultured cells was characterized by FISH using the Peg13-specific digoxin-labeled oligonucleotide probe, as described previously [33]. Briefly, the cells were processed and probed with the Peg13-specific digoxin-labeled oligonucleotide probe or control unrelated probe (1μg/ml), followed by hybridization overnight in a humidified incubator at 37°C. After being washed, the hybrids were reacted with mouse anti-digoxin antibody (1:100, Cell Signaling Technology) and detected with SABC-CY3, followed by nuclearly stained with DAPI. The fluorescent signals were examined under a confocal laser scanning microscope (Leica, Germany).

### Bioinformatic analysis

The position and conservatism of Peg13 in the genome were analyzed using UCSC genome online browsing tool (http://genome.ucsc.edu/). The potential downstream targets of Peg13 and the targets of miR-128-3p were predicted using a StarBase database (http://starbase.sysu.edu.cn), including miRanda, TargetScan, microT, miRmap, PITA, RNA22, and at least four kinds of algorithms and predicted genes were included in subsequent studies. The predicted target genes were uploaded into the online tool Reactome (https://www.reactome.org/) for functional enriched analysis and can be visualized by using GOplot package in R.

### Luciferase assays

The potential roles of miR-128-3p in regulating Peg13 or Sox13-regulated luciferase expression in 293T cells were determined by luciferase assays using the pGL3-luciferase reporter system (Promega, Madison, USA) and Quick Change Site-Directed Mutagenesis kit (Stratagene, La Jolla, USA). The possible binding sequences between Peg13 and miR-128-3p were predicted using online tools, including Starbase and TargetScan. To test the regulatory role of Peg13, the DNA fragment for expression of Peg13 and its mutant for miR-128-3p binding were cloned into pGL3 to generate pGL-Peg13WT and pGL-Peg13 MT, respectively. Similarly, the Sox13 3’ UTR and its mutant for miR-128-3p binding were cloned into pGL3 to generate pGL-Sox13WT and pGL-Sox13MT, respectively. These plasmids were sequenced.

Subsequently, 293T cells were transfected in triplicate with 100 ng pGL-Peg13WT, pGL-Sox13WT, pGL-Peg13 MT or pGL-Sox13MT, together with 10 ng pRL-TK and 50 nm miR-128-3p mimics or control scramble miRNA (miR-128-3p-NC) using Lipofectamine 2000 (ThermoFisher Scientific). Two days later, their luciferase activity was quantified by a Dual-Luciferase Reporter Assay in a Spectra Max M5 microplate reader (Molecular Devices, San Jose, USA). All experiments above were repeated three times.

### Statistics

Data are expressed as mean ± standard error of mean (SEM) from three independent experiments. The difference between groups was statistically analyzed by Student's t test and the comparison among groups was statistically analyzed by one‐way ANOVA. All statistical analyses were performed using SPSS19.0 window (IBM, USA) and the graphs were made using the Prism 6 (GraphPad, CA, USA). *P* < 0.05 was considered statistically significant. *P < 0.05, **P < 0.01 and ***P < 0.001.

## Results

### Identification of mouse embryotic NSCs

**Fig 2:**
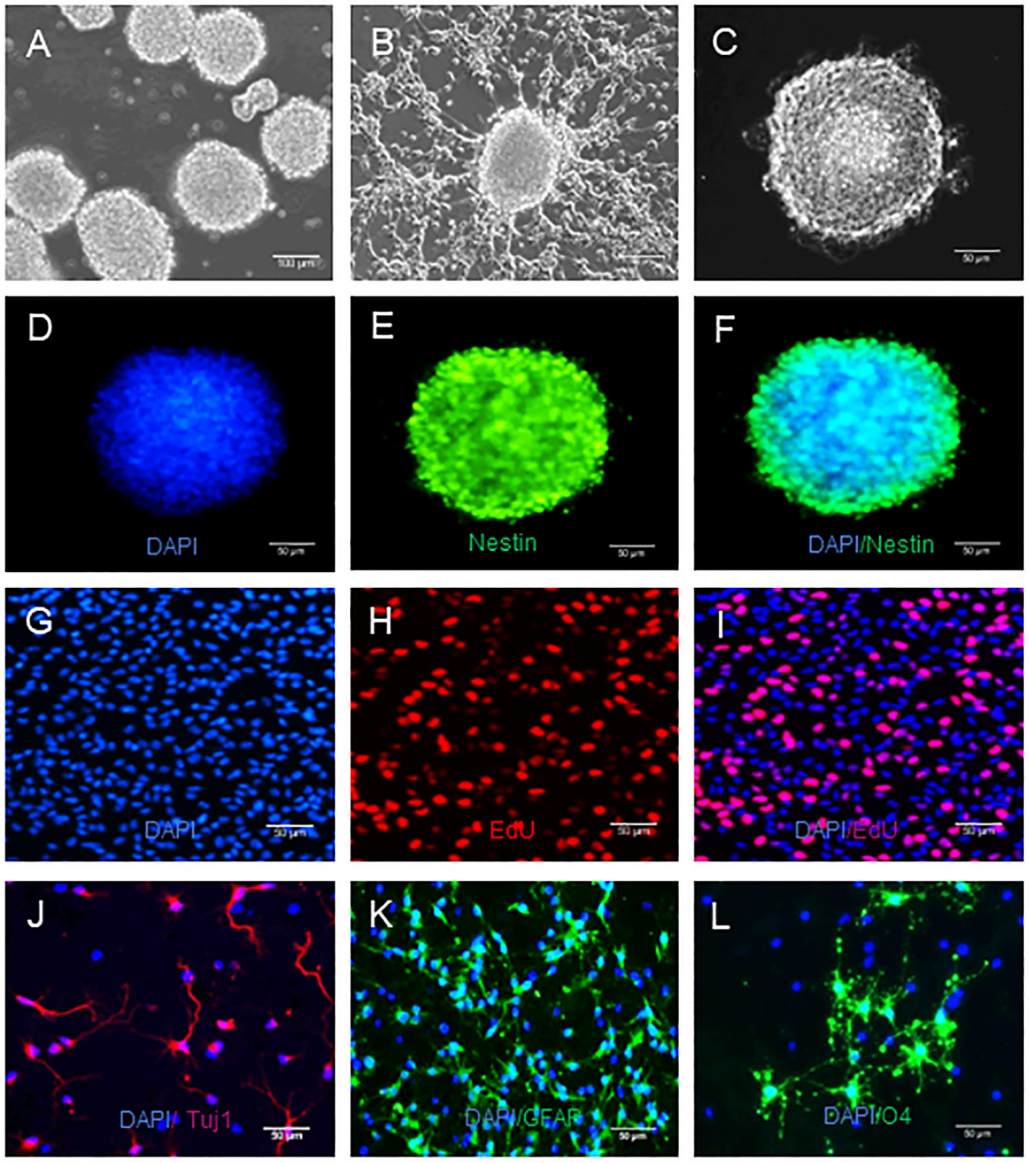
NSCs have the potential of self-renewal and multidirectional differentiation. NSCs were culture for neurosphere formation and differentiation, as described in the method section. The formed neurospheres were imaged and the differentiated neurons, astrocytes and oligodendrocytes were characterized by indirect immunofluorescence. Data are representative images of different types of cells from three separate experiments. A. A microscopic image (magnification 10 x) of typical neurospheres. Scale bar = 100 um. B. The morphology of differentiated cells from NSCs. C. Light microscopy viewed NSCs. Magnification 20 x, Scale bar = 50 um. D-F. Indirect immunofluorescent analysis of neurosphere using anti-Nestin and Alexa Fluor 488-conjugated donkey anti-mouse IgG as well as DAPI. Magnification 20 x, Scale bar = 50 um. G-I. EdU+ cells indicate cell proliferation. Magnification 20 x, Scale bar = 50 um. J-L. Indirect immunofuorescent analysis of differentiated Tuj1+ neurons, GFAP+ astrocytes and O4+ oligodendrocytes using anti- Tuj1, anti-GFAP, anti-O4 and Alexa Fluor 574-labeled donkey anti-rabbit and Alexa Fluor 488-conjugated donkey anti-mouse as well as DAPI, respectively. Magnification 20 x, Scale bar = 50 um.

To characterize the potential toxicity of sevoflurane against NSCs, we isolated mouse embryotic NSCs and found that they formed neurospheres (Fig 2A), expressed NSC-specific marker Nestin (Fig 2C–2F), and displayed strong proliferative ability (Fig 2G–2I). After induction of differentiation (Fig 2B), NSCs differentiated into Tuj1+ neurons (Fig 2J), GFAP+ astrocytes (Fig 2K) and O4+ oligodendrocytes (Fig 2L) These results demonstrated the isolated mouse embryotic NSCs had potent ability of self-renewal and multiple differentiation potentials.

### Sevoflurane exposure reduces self-renewal and differentiation of NSCs in vitro

**Fig 3:**
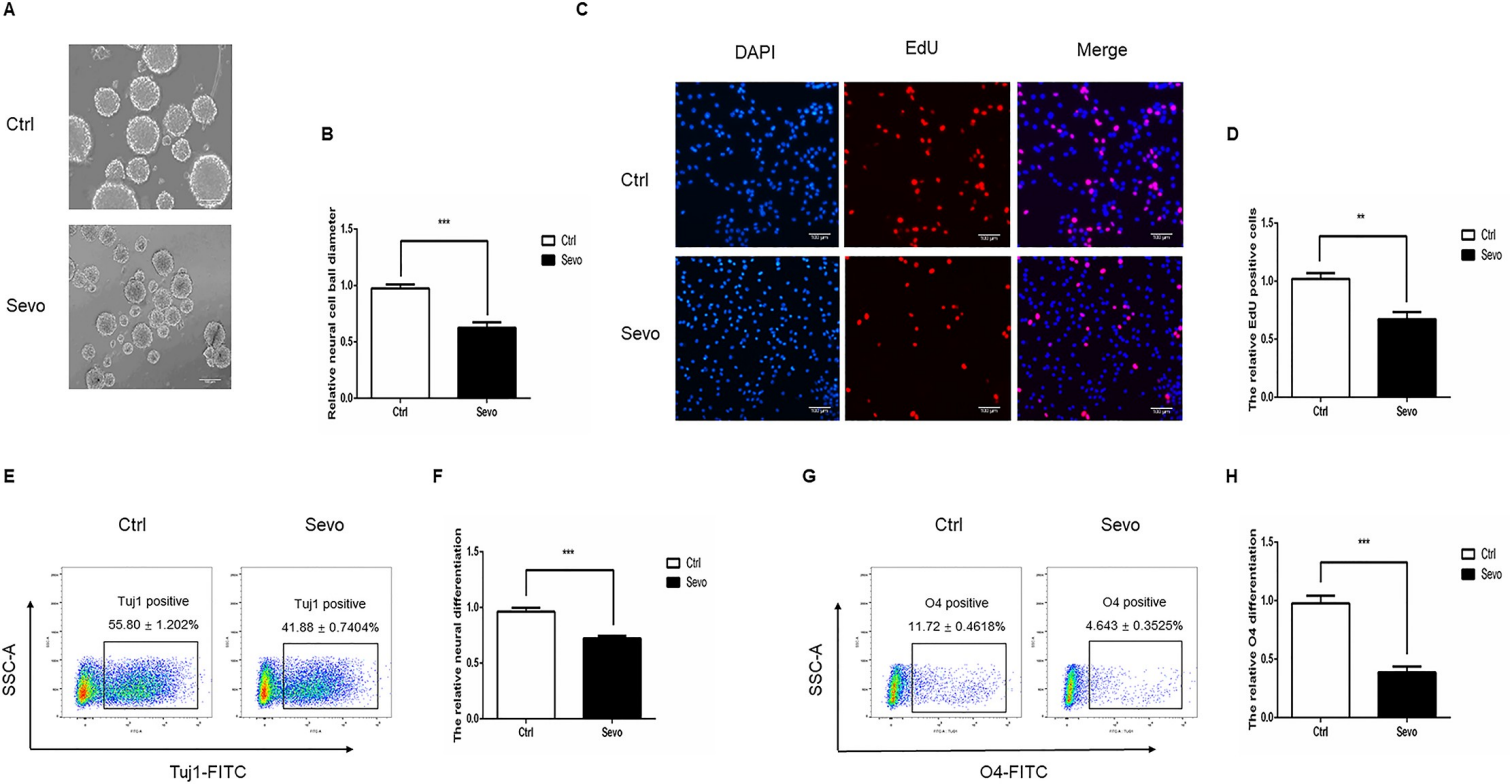
Multiple sevoflurane exposures inhibit the neurosphere formation, proliferation, and differentiation of NSCs. A, B. The neurosphere formation assay indicated that sevoflurane exposure decreased the self-renewal of NSCs. C, D. The EdU assay displayed that sevoflurane exposure reduced the proliferation of NSCs. E, F. Flow cytometry analysis revealed that multiple sevoflurane exposures decreased neuronal differentiation of NSCs. G, H. Multiple sevoflurane exposures inhibited the oligodendrocyte differentiation of NSCs. Data are representative images, charts or expressed as the mean ± SEM of each group of cells from three separate experiments. *P<0.05, **P<0.01, ***P <0.001, determined by Student T test.

To explore the neurotoxicity of repeated sevoflurane exposure, NSCs were exposed to 2 h sevoflurane (4.1% sevoflurane in 60% O_2_) or control O_2_ alone for three consecutive days, and their self-renewal was analyzed by neurosphere formation and EdU assays. In comparison with the control NSCs, sevoflurane exposure significantly decreased the sizes of formed neurospheres (Fig 3A and 3B) and EdU incorporation in NSCs (Fig 3C and 3D). Following induction of differentiation, flow cytometry analysis indicated that sevoflurane exposure significantly reduced the frequency of β-tubulin-III+ neurons (Fig 3E and 3F) and O4+ oligodendrocytes (Fig 3G and 3H). Together, such data indicated that sevoflurane exposure significantly inhibited the self-renewal and differentiation of mouse NSCs in vitro.

### Sevoflurane inhibits the self-renewal and differentiation of NSCs by downregulating Peg13 expression

**Fig 4:**
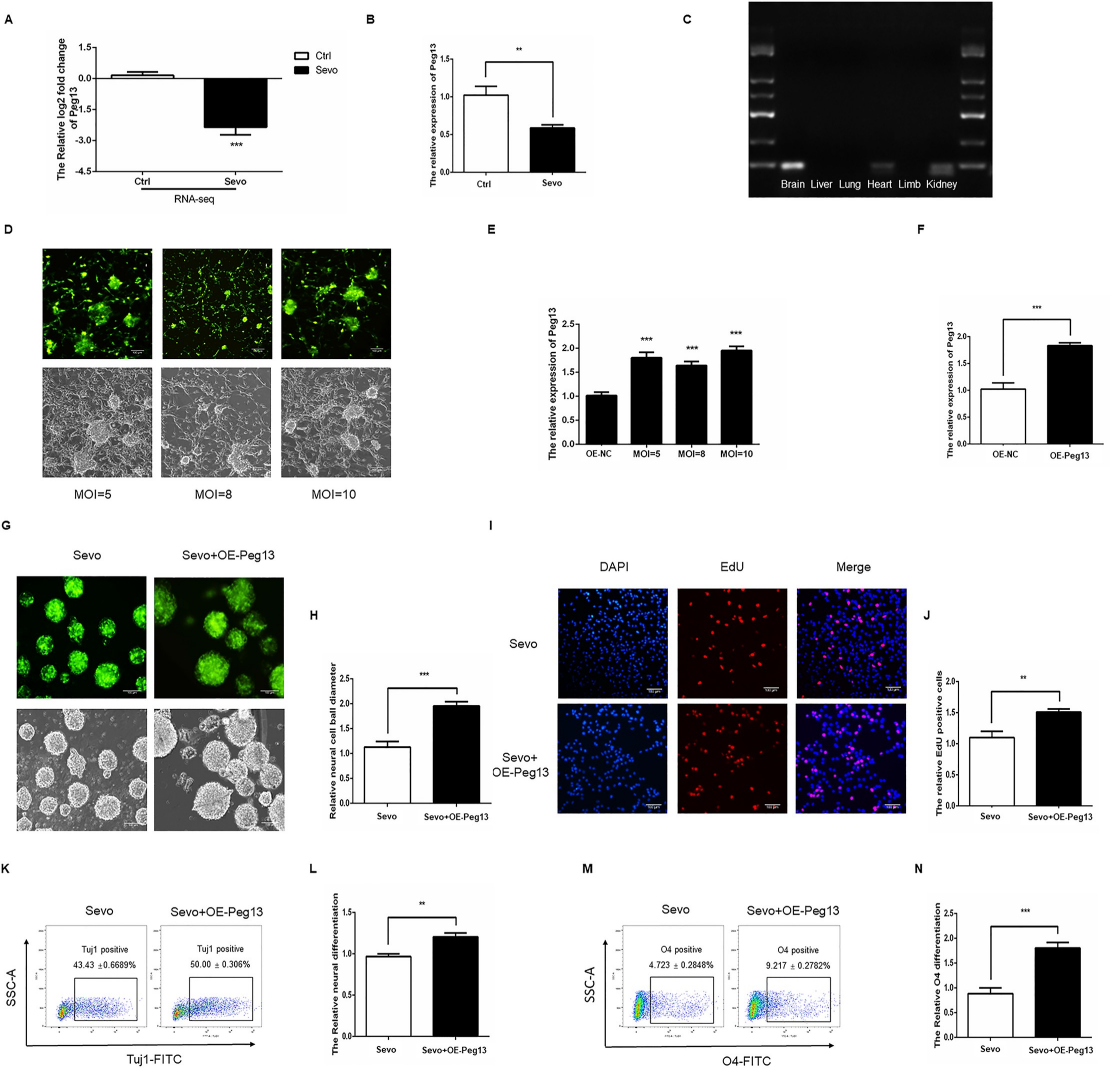
Peg13 attenuates the sevoflurane-induced NSC damage. A. RNAseq assay indicated that sevoflurane significantly reduced Peg13 transcripts in NSCs. B. Quantitative RT-PCR revealed that sevoflurane significantly reduced Peg13 transcripts in NSCs. C. Agarose gel electrophoresis assay displayed high levels of Peg13 transcripts in the brain of mice. D, E. Transduction with different doses of OE-Peg13 lentivirus for Peg13 expression increased Peg13 transcripts in NSCs a dose-dependent manner, determined by fluorescent microscopy and qRT-PCR. F. Transduction with OE-Peg13 at MOI of 10 increased Peg13 transcripts by 1.8 folds, determined by qRT-PCR. G, H. Peg13 over-expression recovered the sevoflurane-decreased neurosphere formation in NSCs. I, J. Peg13 over-expression restored the sevoflurane-decreased EdU incorporation in NSCs. K-N. Flow cytometry analyses revealed that Peg13 over-expression promoted the neuronal and oligodendrocyte differentiation in the sevoflurane-exposed NSCs. Data are representative images, charts or expressed as the mean ± SEM of each group of cells from three separate experiments. *P<0.05, **P<0.01, ***P <0.001, determined by Student T test.

After sevoflurane exposure, we analyzed lncRNA expression in NSCs by RNAseq. We found that sevoflurane exposure significantly reduced the lncRNA Peg13 transcripts in NSCs (Fig 4A). Further qRT-PCR demonstrated that sevoflurane exposure decreased Peg13 transcripts by more than 50% (Fig 4B) and agarose electrophoresis revealed that the Peg13 was highly expressed in the brain of mice (Fig 4C). Next, we established Peg13 stable NSCs by lentivirus-related technology and we found that transduction with OE-Peg13 lentivirus increased Peg13 over-expression in a dose-dependent manner, determined by fluorescent inverted phase contrast microscope and qRT-PCR (Fig 4D and 4E). To understand the regulatory role of Peg13, we packaged lentivirus for Peg13 over expression.The multiplicities of infection (MOI) of transfection were determined by fluorescent inverted phase contrast microscope and transduction with OE-Peg13 at a MOI of 10 increased Peg13 transcripts by 1.8 times, which was used for subsequent studies (Fig 4F). Neurosphere formation assays indicated that Peg13 over-expression mitigated the sevoflurane-decreased neurosphere formation (Fig 4G and 4H) and EdU incorporation of NSCs (Fig 4I and 4J). Flow cytometry displayed that Peg13 over-expression also minimized the sevoflurane-inhibited neuronal (Fig 4K and 4L) and oligodendrocyte (Fig 4M and 4N) differentiation of NSCs in vitro (Fig 4M and 4N). Such data indicated that sevoflurane exposure inhibited the self-renewal and differentiation of NSCs by down-regulating Peg13 expression.

### Peg13 attenuates the sevoflurane-inhibited NSC self-renewal and differentiation by sponging miR-128-3p

**Fig 5:**
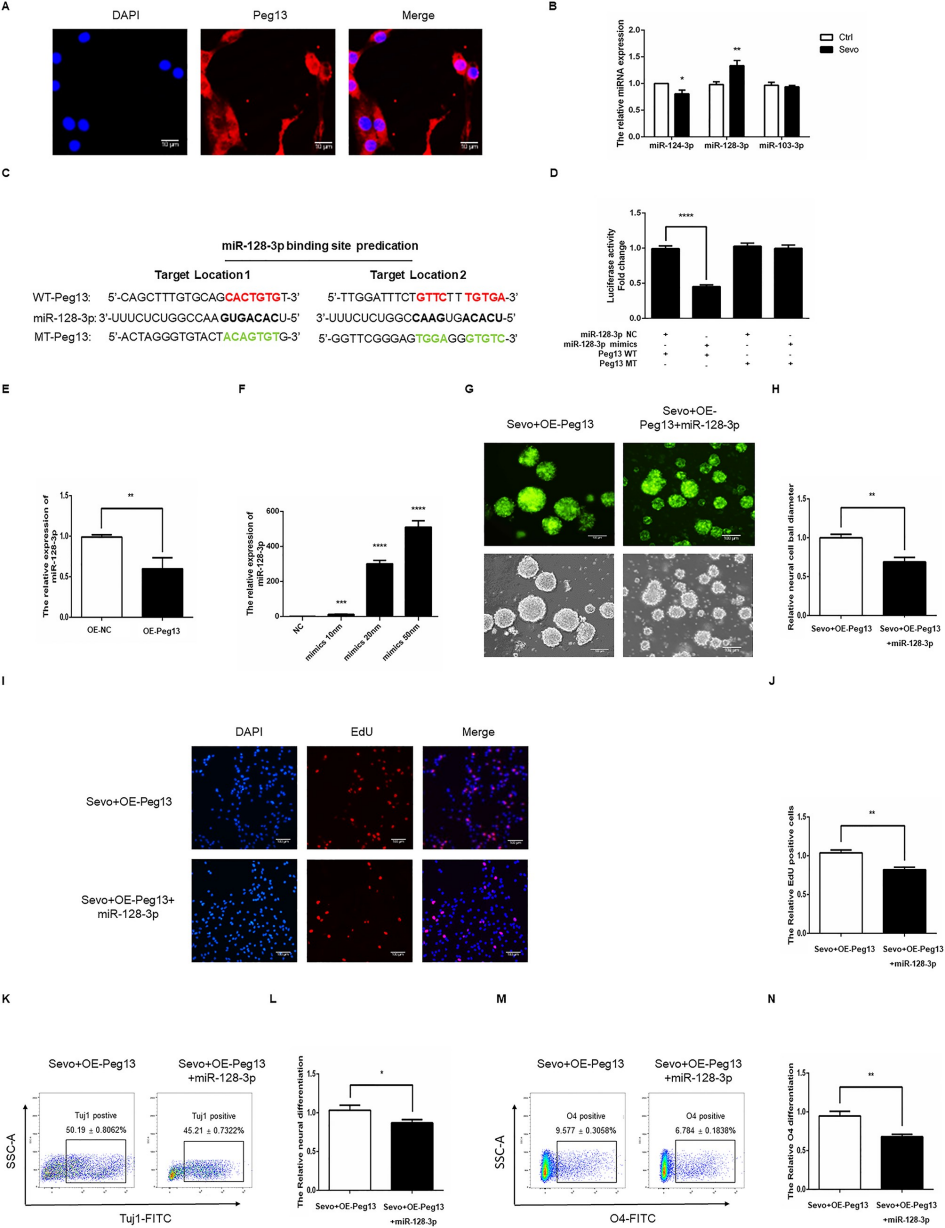
Peg13 mitigates the effect of sevoflurane by targeting miR-128-3p in NSCs. A. FISH identified the cytoplasmic and nuclear distribution of Peg13 transcripts in NSCs. Magnification 100 x, Scale bar = 10 um. B. Quantitative RT-PCR revealed that sevoflurane exposure increased miR-128-3p transcripts in NSCs. C. Bioinformatics predicted the potential interaction between miR-128-3p with Peg13. D. Luciferase reporter assay indicated that miR-128-3p inhibited the Peg-13-regualted, but not its mutant-regulated, luciferase expression in 293T cells. E. Peg13 over-expression decreased miR-128-3p transcripts in NSCs. F. Transfection with different doses of miR-128-3p mimics induced miR-128-3p transcripts in NSCs in a dose-dependent manner, determined by qRT-PCR. G, H. Induction of miR-128-3p expression reduced neurosphere formation in the sevoflurane-exposed Peg13-over-expressed NSCs. J, K. Induction of miR-128-3p expression decreased EdU incorporation in the sevoflurane-exposed Peg13-over-expressed NSCs. L, O. Induction of miR-128-3p expression inhibited neuronal and oligodendrocyte differentiation of the sevoflurane-exposed Peg13-over-expressed NSCs. Data are representative images, charts or expressed as the mean ± SEM of each group of cells from three separate experiments. *P<0.05, **P<0.01, ***P <0.001, determined by Student T test.

To understand the regulatory role of Peg13, we identified Peg13 expression by FISH and found that the Peg13 was expressed predominantly in the cytoplasm of NSCs (Fig 5A). Given that lncRNA can sponge miRNAs we determined the effect of sevoflurane exposure on the expression levels of miRNAs by qRT-PCR. As shown in Fig 5B, sevoflurane exposure significantly up-regulated miR-128-3p expression in NSCs. Bioinformatic analysis predicted that miR-128-3p bound to the Peg13 (Fig 5C). Luciferase assays revealed that transfection with miR-128-3p mimics, but not the control, significantly mitigated the Peg13-regulated luciferase expression in 293T cells (Fig 5D). However, miR-128-3p did not significantly alter the mutated Peg13-regulated luciferase expression in 293T cells. Interestingly, induction of Peg13 over-expression significantly decreased miR-128-3p transcripts in NSCs (Fig 5E). Transfection with different doses of miR-128-3p mimics induced miR-128-3p expression in a dose-dependent manner (Fig 5F). We chose 50 nm miR-128-3p for subsequent experiments. In comparison with that in the scramble miRNA-transfected sevoflurane-exposed Peg13 over-expressed cells, transfection with miR-128-3p mimics significantly decreased neurosphere formation (Fig 5G and 5H), EdU incorporation (Fig 5I and 5J), neuronal (Fig 5K and 5L) and oligodendrocyte differentiation of NSCs (Fig 5M and 5N). Collectively, such data pointed that Peg13 attenuated the sevoflurane exposure-inhibited NSC self-renewal and differentiation by sponging miR-128-3p in vitro.

### Peg13 sponges miR-128-3p to preserve Sox13 expression in sevoflurane-exposed NSCs

**Fig 6:**
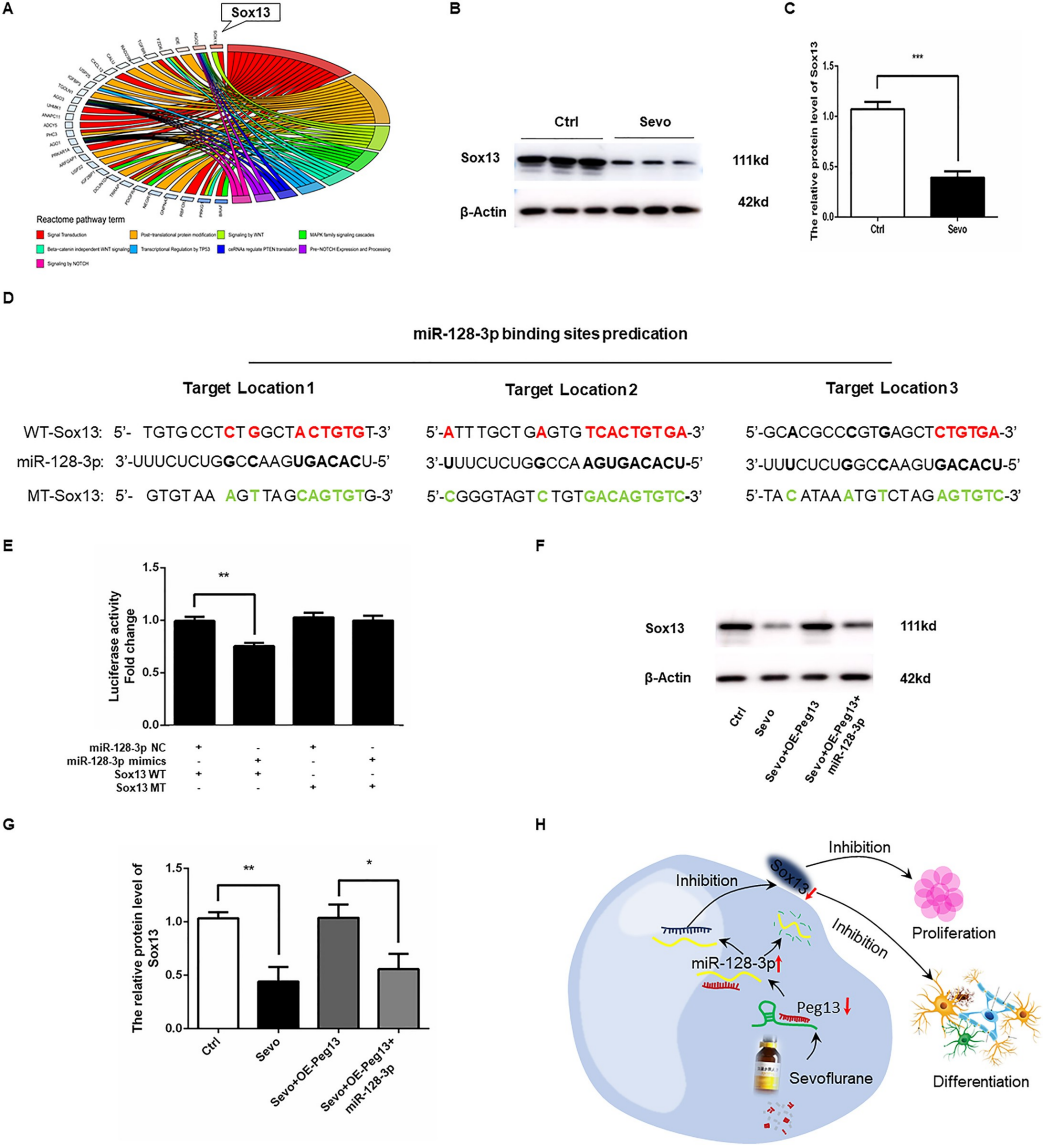
Peg13 over-expression rescues the sevoflurane neurotoxicity by sponging miR-128-3p to preserve Sox13 expression in NSCs. A. Pathway analysis indicated that the Sox13-related signal pathways were involved in the sevoflurane-related neurotoxicity in NSCs. B, C. Western blotting displayed that sevoflurane exposure decreased Sox13 expression in NSCs. D. Bioinformatics predicted that miR-128-3p bound to the 3′UTR of Sox mRNA. E. Luciferase reporter assay indicated that miR-128-3p over-expression reduced the wild-type Sox13-regulated, but not its mutant-regulated, luciferase expression in 293T cells. F, G. Peg13 over-expression restored Sox13 expression in the sevoflurane-exposed NSCs, which was mitigated by miR-128-3p over-expression. H. A diagram illustrates the mechanisms underlying the regulatory role of the Peg13/miR-128-3p/Sox13 axis in sevoflurane-related neurotoxicity in NSCs. Data are representative images, or expressed as the mean ± SEM of each group of cells from three separate experiments. *P<0.05, **P<0.01, ***P <0.001, determined by Student T test or ANOVA.

The Sox genes are crucial for the early embryonic development and neurogenesis and the Sox13 related signal pathways were involved in the sevoflurane-related neurotoxicity (Fig 6A). To further understand the neurotoxicity of sevoflurane, we measured the Sox13 expression in NSCs and discovered that sevoflurane exposure significantly decreased the Sox13 protein expression in NSCs (Fig 6B and 6C). Bioinformatic analysis predicted that miR-128-3p bound to the 3’ UTR of Sox13 (Fig 6D). Luciferase report assay revealed that transfection with miR-128-3p mimics, but not the control scramble miRNA, inhibited the Sox13-regulated luciferase expression in 293T cells, but did not affect the Sox13 mutant-regulated luciferase expression in 293T cells (Fig 6E), suggesting that miR-128-3p targeted the Sox13 expression. Finally, while sevoflurane exposure significantly decreased Sox13 expression, which was abrogated by Peg13 over-expression, induction of miR-128-3p expression abrogated the effect of Peg13 to reduce Sox13 expression in NSCs (Fig 6F and 6G). Collectively, such data indicated that Peg13 attenuated the sevoflurane exposure-inhibited Sox13 expression by sponging miR-128-3p, reducing sevoflurane exposure-related neurotoxicity against NSCs (Fig 6H).

## Discussion

Sevoflurane has been widely used for anesthesia in the clinic. Previous studies have shown that exposure to sevoflurane in early life can cause neuronal defects in human and animals [1, 4, 9]. In this study, we found that sevoflurane exposure inhibited the self-renewal and differentiation of mouse embryotic NSCs. These findings were consistent with previous observations with other anesthetic drugs [18, 25] and support the notion that exposure to anesthesia in early life can inhibit neurogenesis and cause neurological impairments [1, 3]. Although the central nervous system, particularly for the brain in humans and rodents, usually undergoes continual remodeling in a period after birth it may be safer to avoid unnecessary anesthesia and surgery during the neurogenesis and early life of a person and may have profound regulation effect to the nervous system through different pathophysiological processes. The main objective of our research was to determine the influence of sevoflurane on neurogenesis of NSCs, and to explore its comprehensive downstream mechanisms. Our results clearly reveal that sevoflurane can inhibit the neurosphere formation, proliferation and differentiation of NSCs. Our study is the first to demonstrate that the multiple sevoflurane exposures decrease the expression of Peg13 in the NSCs, leading to the inhibition of neural differentiation through the chain reaction of miR-128-3p and Sox13. Our findings clearly show the significant role of Peg13 in the differentiation of NSCs, and elaborate the mechanisms underlying sevoflurane toxicity.

LncRNAs can sponge its target miRNAs to preserve the expression of miRNA-targeted genes to regulate neurogenesis besides modulating its targeted transcription factors [34]. In this study, we uncovered that sevoflurane exposure significantly decreased lncRNA Peg13 and Sox13 expression, but increased miR-128-3p expression in NSCs. More importantly, lncRNA Peg13 significantly mitigated the sevoflurane exposure-related neurotoxicity against mouse embryotic NSCs by sponging miR128-3p to preserve Sox13 expression. Such novel findings extended previous observations that lncRNA Gm15621 over-expression alleviates sevoflurane-related neurotoxicity by inhibiting the miR-133a/Sox4 axis [35]. Hence, our findings may provide new insights into the pathogenesis of sevoflurane-related neurotoxicity. Our findings, together with facts that sevoflurane exposure significantly up-regulated lncRNA Gadd45a expression and lncRNA HOTAIR in rat hippocampus [25, 36], but down-regulated LncRNA Malat1 expression in infant rats [37], suggest that different lncRNAs may have varying functions and form a network in regulating the sevoflurane-related neurotoxicity against NSCs. This regulatory network will determine the self-renewal and differentiation of NSCs. We are interested in further investigating whether and how these lncRNAs can synergistically and antagonistically regulate the sevoflurane-related neurotoxicity.

Many studies have indicated that miRNAs are crucial for regulating diverse physiological and pathological processes, such as differentiation, apoptosis, proliferation and cancer [38]. MiRNA usually exerts its biological function through binding the 3’UTR of its targeted genes to suppress their expression [39, 40]. MiR-128 is an important molecule in the CNS and has been shown to regulate multiple processes, such as neurogenesis, neural differentiation and neuronal migration, outgrowth and intrinsic excitability [41, 42]. MiRNA-128-3p is a mature miRNA in the 3' sequence of mir-128 stem loop structure. Previous studies have shown that miR-128-3p can promote the differentiation of intramuscular adipocyte differentiation [43] and Sox13 is a transcription factor of the SRY-related HMG-box family, regulating embryotic development [44]. Sox13 can form a regulatory complex with other members in the family to determine the cell fate and is crucial for oligodendrocyte development [45]. We found that Sox13 expression was down-regulated after sevoflurane exposure and was targeted by miR-128-3p in NSCs. Such data extended previous observation that miR-138-5p targeted Sox13 to regulate angiogenesis in Glioma [46]. Thus, multiple miRNAs may target the same gene mRNA to regulate its expression during the process of neurogenesis.

However, our study still has limitations, including the lack of in vivo study and precise mechanisms by which sevoflurane exposure down-regulated lncRNA Peg13 expression in NSCs. Our findings may provide new insights into the sevoflurane-related neurotoxicity and highlight the importance of the Peg13/miR-128-3p/Sox13 axis in regulating neurogenesis.

In conclusion, we demonstrated the vital role of Peg13 in regulating neural differentiation and further elaborated the miR-128-3p-Sox13-associated molecular mechanisms, which could open new revenues to further investigate the role of lncRNAs on neurogenesis.

## Supporting information

S1 Raw images (PDF)
