# Supplementary figures and images for: Long non-coding RNA Peg13 attenuates the sevoflurane toxicity against neural stem cells by sponging microRNA-128-3p to preserve Sox13 expression
Source: PLoS One. 2020 Dec 9;15(12):e0243644. doi: 10.1371/journal.pone.0243644 (PMC7725402; doi:10.1371/journal.pone.0243644)

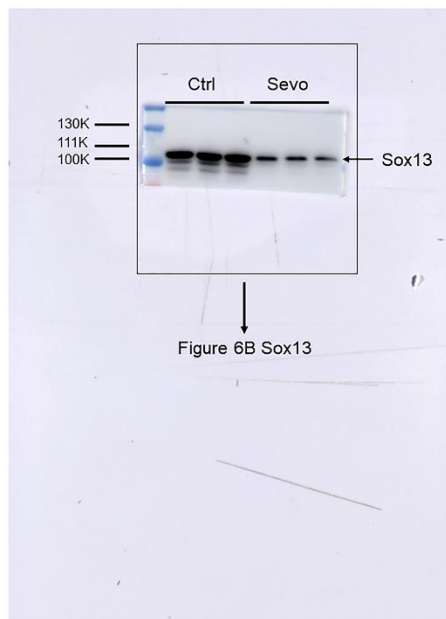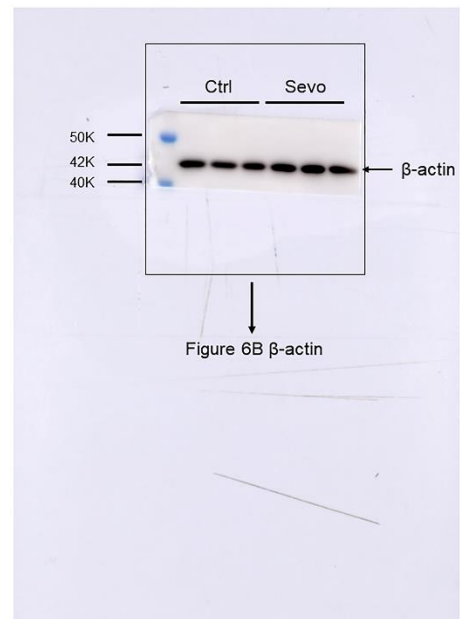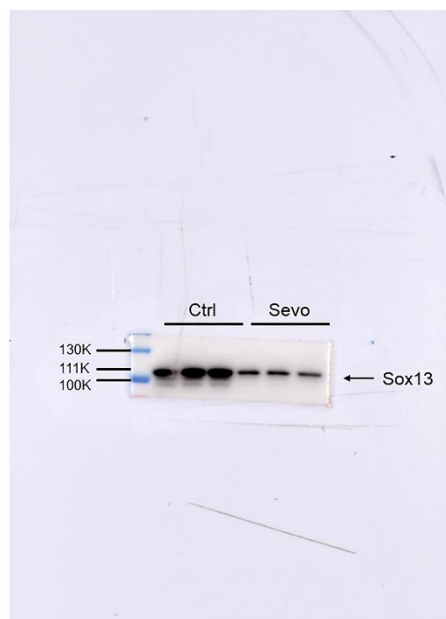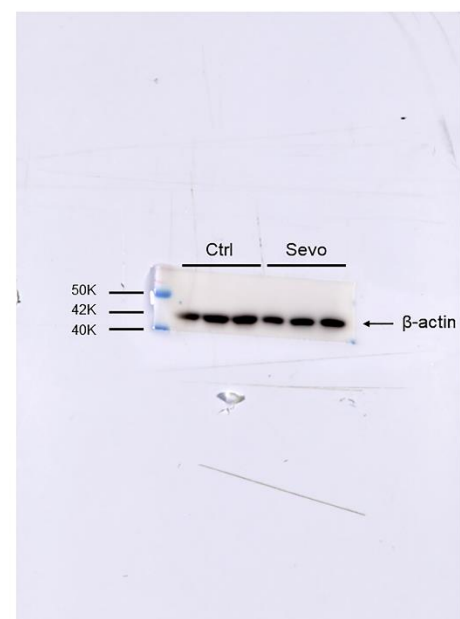

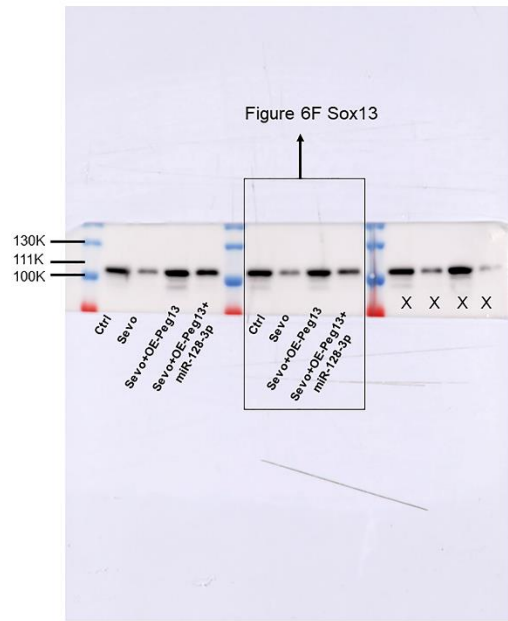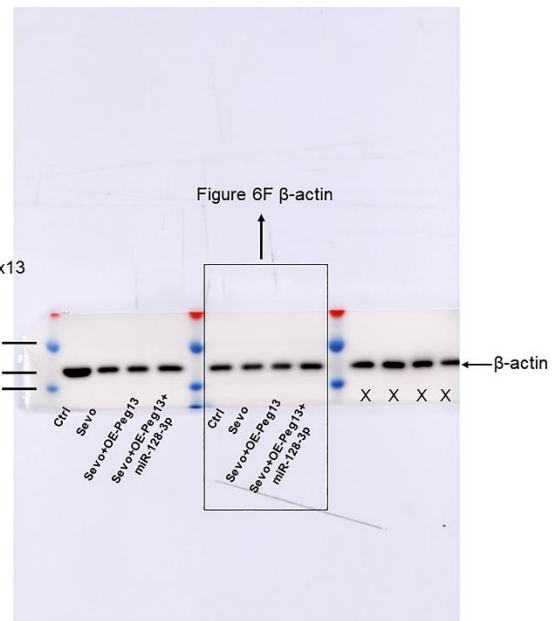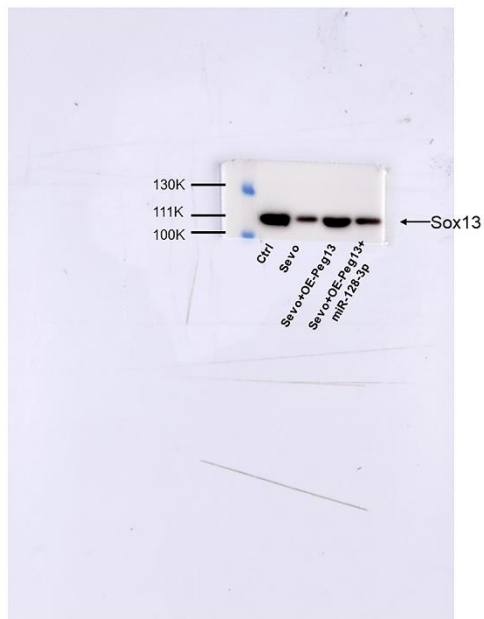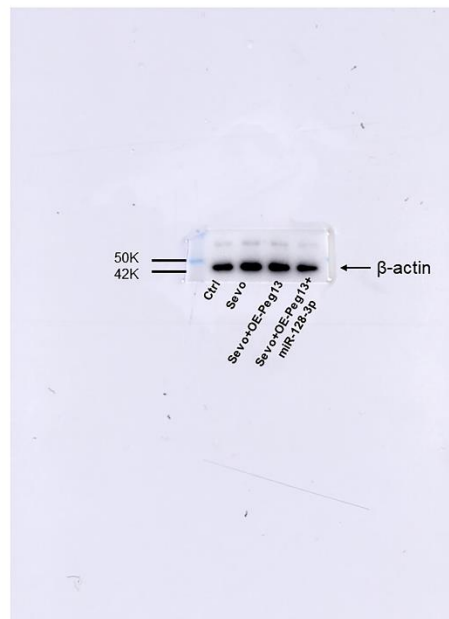

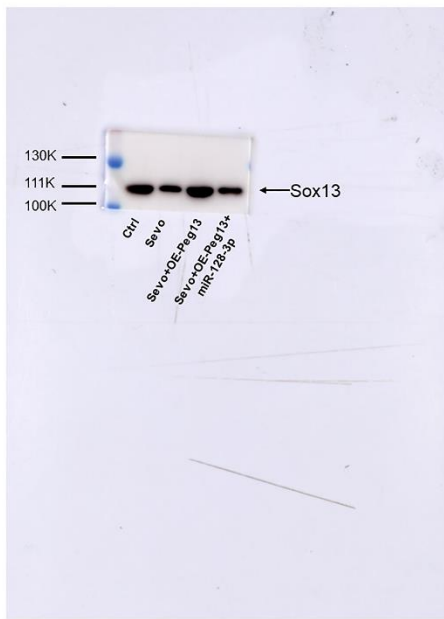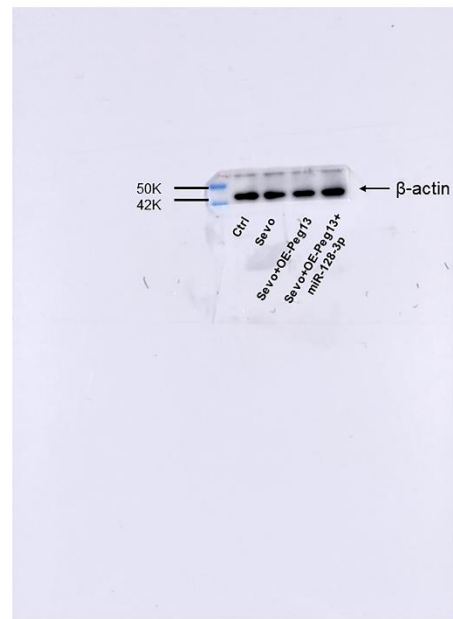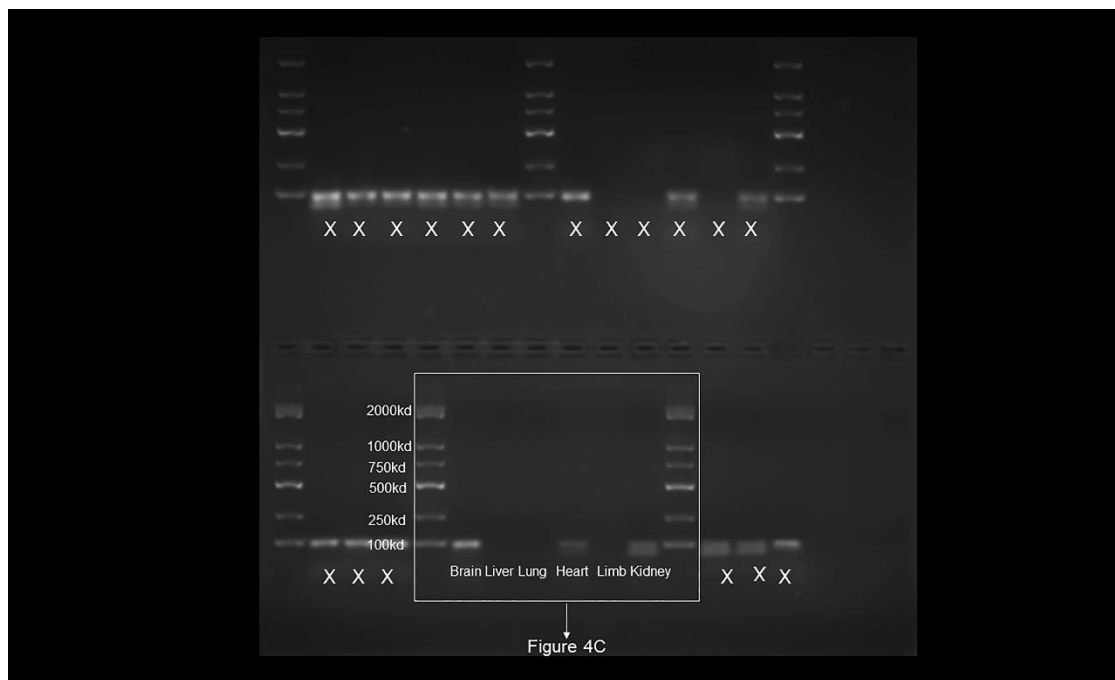

Figure 4C

Supplement: S1 Raw images — (PDF) [file pone.0243644.s001.pdf]
